# Considerations for de-escalating universal masking in healthcare centers

**DOI:** 10.1017/ash.2023.200

**Published:** 2023-07-26

**Authors:** Caroline Landelle, Gabriel Birgand, James R. Price, Nico T. Mutters, Daniel J. Morgan, Jean-Christophe Lucet, Solen Kerneis, Walter Zingg

**Affiliations:** 1 University of Grenoble Alpes, CNRS, UMR 5525, Grenoble INP, CHU Grenoble Alpes, Infection Prevention and Control Unit, 38000 Grenoble, France; 2 National Institute for Health Research Health Protection Research Unit in Healthcare Associated Infections and Antimicrobial Resistance at Imperial College London, London, UK; 3 Regional Center for Infection Prevention and Control Pays de la Loire, Centre Hospitalier Universitaire de Nantes, Nantes, France; 4 Brighton and Sussex Medical School, Brighton, UK; 5 Institute for Hygiene and Public Health, University Hospital Bonn, Bonn, Germany; 6 University of Maryland School of Medicine, Baltimore, MD, USA; 7 VA Maryland Healthcare System, Baltimore, MD, USA; 8 Infection Control Unit, Hôpital Bichat-Claude Bernard, Assistance Publique-Hôpitaux de Paris, Paris, France; 9 Department of Infectious Diseases and Hospital Epidemiology, University Hospital of Zurich, Zurich, Switzerland

**Keywords:** COVID-19, healthcare setting, infection control, universal masking, medical face mask, respiratory viral infections

## Abstract

Three years after the beginning of the COVID-19 pandemic, better knowledge on the transmission of respiratory viral infections (RVI) including the contribution of asymptomatic infections encouraged most healthcare centers to implement universal masking. The evolution of the SARS-CoV-2 epidemiology and improved immunization of the population call for the infection and prevention control community to revisit the masking strategy in healthcare. In this narrative review, we consider factors for de-escalating universal masking in healthcare centers, addressing compliance with the mask policy, local epidemiology, the level of protection provided by medical face masks, the consequences of absenteeism and presenteeism, as well as logistics, costs, and ecological impact. Most current national and international guidelines for mask use are based on the level of community transmission of SARS-CoV-2. Actions are now required to refine future recommendations, such as establishing a list of the most relevant RVI to consider, implement reliable local RVI surveillance, and define thresholds for activating masking strategies. Considering the epidemiological context (measured via sentinel networks or wastewater analysis), and, if not available, considering a time period (winter season) may guide to three gradual levels of masking: (i) standard and transmission-based precautions and respiratory etiquette, (ii) systematic face mask wearing when in direct contact with patients, and (iii) universal masking. Cost-effectiveness analysis of the different strategies is warranted in the coming years. Masking is just one element to be considered along with other preventive measures such as staff and patient immunization, and efficient ventilation.

## Introduction

With the emergence of the pandemic, wearing face masks became one of the most prominent interventions against COVID-19^1^. In the absence of protective immunity, many countries made mask wearing mandatory for the general population in crowded areas where social distancing could not be maintained (such as public transport), and recommended wearing them even outdoors^2^. Before the COVID-19 pandemic, wearing a medical face mask was recommended for healthcare professionals (HCPs) when caring for patients with respiratory symptoms as part of standard precaution measures and for patients with respiratory symptoms as part of the respiratory etiquette. Transmission-based precautions were also recommended for HCPs in close contact with symptomatic patients as part of droplet precautions.

Early in the pandemic, transmission of SARS-CoV-2 by asymptomatic and presymptomatic COVID-19 cases appeared critical in the epidemiology and control of the disease^3^. Universal face masking in hospitals is usually defined as the requirement to wear a medical or surgical mask by all staff (clinical and nonclinical), patients, and visitors at any time. In the early phase of the pandemic, universal masking was part of guidelines on respiratory protection, issued by leading organizations in infection prevention and control (IPC)^4^. Following Hong Kong, Singapore, and other regions of Asia, the United States and Germany introduced universal masking in March 2020 as part of a range of non-pharmaceutical measures to control the spread of SARS-CoV-2 in healthcare settings. This change was unprecedented since, prior to the COVID-19 pandemic, studies had not shown an advantage of masks to prevent acquiring or spreading of respiratory pathogens in HCPs by the use of medical face masks. Three years later, the lower impact of COVID-19 on morbidity and mortality, fatigue of HCPs to wear personal protective equipment (PPE), cost, and ecological considerations have challenged the benefits of universal masking, and called for discussions on de-implementation.

In this context of evolving knowledge and recommendations, we reflected on the factors to consider de-escalating universal masking in healthcare centers.

### Within-hospital SARS-CoV-2 risk of transmission

Our understanding of within-hospital transmission of respiratory viruses remains imperfect, despite increased attention over the last three years. Using viral genomics to investigate sources of SARS-CoV-2 transmission in HCPs, the majority of infections (57.9%) could not be linked to a patient or coworker^5^. Only 10.5% and 4.2% of infections were traced to a coworker or a patient, respectively. A UK study combined viral genomic and epidemiological data in 2181 patients and HCPs^6^. During the first wave with an increasingly strict masking policy (“when in contact with suspected cases”, followed by “all patient contact”, and finally by “universal masking”), transmissions were staff-to-staff in 31.6%, patient-to-patient in 27.1%, patient-to-staff in 25.5%, and staff-to-patient in 15.5%. During the second wave with universal masking from the beginning, transmissions were patient-to-patient in 52.1%, patient-to-staff in 21.2%, staff-to-patient in 13.5%, and staff-to-staff in 12.9%. A staff member was identified as the index case in 50.6% of the transmission chains in the first wave but only in 31.3% in the second wave. The authors concluded that intensified control measures during the pandemic likely reduced transmission between healthcare workers but not between patients. The analysis of data from 11,290 patients admitted to shared rooms revealed that almost 40% of exposed patients were getting infected^7^.

### Current evidence on the effectiveness of universal masking to prevent the transmission of COVID-19 and other respiratory viral infections

The effectiveness of universal masking on the transmission of respiratory viruses was studied already before the pandemic. Wearing a medical mask or N95 respirator throughout work shifts by HCPs significantly reduced self-reported clinical respiratory illness (risk ratio [RR] = 0.59; 95% CI: 0.46–0.77) and influenza-like illness (RR = 0.34; 95% CI: 0.14–0.82)^8^. Systematic masking of all individuals in direct contact with stem cell transplant recipients significantly reduced infections due to respiratory infections in patients^9^. A universal masking policy in a neonatal unit reduced the number of infections due to respiratory viruses from 38 during 2011–2015 to 5 during 2017–2019, with respiratory syncytial viruses (RSV), parainfluenza viruses, and rhinoviruses representing two-thirds of the cases^10^.

In March 2020, a large US hospital implemented a prevention strategy combining systematic testing of symptomatic HCPs and universal masking for both HCPs and patients^11^. Before universal masking, the SARS-CoV-2 positivity ratio increased exponentially from 0% to 21.3%, with an average increase of 1.2% per day. During universal masking by HCPs and patients, the positivity rate decreased linearly from 14.7% to 11.5%, with an average decline of 0.5% per day. Contact tracing of 226 patients exposed to HCPs with confirmed COVID-19 identified only one possible transmission by an unmasked HCP prior to universal masking^12^. In the same study population, only 1 of 12 hospitalized patients testing positive on hospital day 3 or later was considered to have hospital-acquired COVID-19^13^. The authors concluded that there was no convincing evidence of in-hospital transmission after the implementation of universal masking for both staff and patients. During the first winter season of the COVID-19 pandemic, Europe and the US experienced a quasi-absence of seasonal influenza^14^. Epidemiological investigations and ecological studies provided convincing evidence that universal non-pharmaceutical interventions in the general population, including universal masking, were associated with relevant reductions of non-COVID-19 RVIs^15^. The impact of universal masking on the transmission of COVID-19 and non-COVID-19 RVIs was confirmed by other studies^16–20^.

Together, these findings suggest that universal masking impacts on overall hospital transmission of respiratory viruses, particularly during periods of high community prevalence^21^. However, most of these studies used a before-after design, with limited control for confounders and without sensitivity analysis on time periods.

### Factors to consider for de-escalating or maintaining universal masking in healthcare centers

Lessons learned in healthcare centers during the COVID-19 pandemic, and particularly the evolving understanding of respiratory virus transmission, should drive the way medical face masks are used in the future. Importantly, universal masking is only one measure to reduce the risk of transmission of respiratory viruses among other non-pharmaceutical interventions such as hand hygiene, room ventilation, testing strategies, distancing, and patient/visitor restrictions. Some factors support de-escalating universal masking but others are in favor of maintaining it (Table 1).

**Table 1.** Factors for and against De-escalating or Maintaining Universal Masking in Healthcare Centers

Targeted population	De-escalating universal masking	Maintaining universal masking
HCP and patient perspective	**Adherence** Lack of adherence and compliance with universal masking related to fatigue, discomfort, and tolerability	Rare hospital transmission with good adherence and compliance of universal masking policy
	**Epidemiology** Decreasing benefit of universal masking in healthcare settings during low community transmission	Policy driven by imperfect epidemiological data (no real-time data, testing bias); challenge of back-and-force reinstitution of universal masking
	**Immunity and treatment options** High level of vaccine and infection-induced immunity and availability of effective treatment and prevention tools	Vaccine hesitancy and waning immunity
	**Community measures** Inconsistencies with non-pharmaceutical measures in the population	Prevention of transmission by asymptomatic and presymptomatic individuals; anticipating the occurrence of variants or emerging respiratory viruses
	**Cost and logistics** Rupture of supply chains, high cost, and ecological concerns	Counterbalancing costly installation of ventilation systems or investments to improve infrastructure
HCP perspective	**Absenteeism and presenteeism** Universal masking applying to the occupational setting only	Absenteeism due to occupational transmission of respiratory viruses; presenteeism
	**Staff without patient contact** Unclear benefit for HCP without direct patient contact	
Patient perspective	Improved HCP-patient relationship in the absence of face covering	Protection of vulnerable patients

Note. HCPs: healthcare professionals.

#### Provider’s adherence and compliance with universal masking

High compliance and correct use of masks are important to ensure the effectiveness of universal masking. Experiments simulating the transmission of SARS-CoV-2 between two individuals demonstrated that source control alone is more effective than protection of the exposed individual alone, but that protection is most effective when both sides wear a mask, which makes the case of universal masking^22^. The main factor affecting the risk of transmission in the universal masking scenario is the leakage between the mask and the face^23^. Correct donning of face masks was 88% and 70% in COVID-19 and non-COVID-19 wards of a German University hospital, respectively^24^. Although the COVID-19 wards performed better, deficits of correct use of respirators were important. During a 4-week period after the implementation of a mandated universal masking policy in a tertiary-care center in the US, the median weekly face-mask compliance was 82.2% (range: 80.8–84.4%)^25^. Compliance increased to 92.6% (84.6%-97.9%) after implementing audits, passive feedback, active discussion, and better communication by leadership.

Adherence with face mask use by HCPs relies on organizational (communication, support from managers, workplace culture, training, and access to face masks) and individual factors (trust in face masks, desire to deliver good patient care, and feelings of safety from exposure to COVID-19 with and without vaccination)^26^. In a survey among 1,109 HCPs from an academic medical center with universal masking, only 33% of participants reported face masks being comfortable^27^. The main reported disadvantages of face mask wearing were breathing difficulties (80%), heat sensation (68%), and skin irritation (46.2%). In addition, only 17.1% felt that speech was sufficiently audible by others. During observations performed across the vaccination campaigns, HCPs reported feeling safe from exposure to COVID-19 when being around their colleagues^28^. Concerns about the negative impact of face masks on the physician-patient relationship were raised repeatedly. A study in surgery reported that patients appreciated to see the face of their surgeons when using transparent face masks, improving communication and trust, and giving a perception of increased empathy^29^.

#### Impact of community transmission on the efficacy of universal masking in healthcare centers

A point of attention is the level of exposure during both occupational and nonoccupational activities. Nonoccupational exposure plays a critical role in the risk of COVID-19 among HCPs. A meta-analysis showed a higher risk of COVID-19 for HCPs exposed outside the workplace (14%–32%) in comparison with exposure inside (6%–13%)^30^. Using survey data on self-reported risk factors, staff with confirmed household contacts were at highest risk of COVID-19 (adjusted Odds Ratio [aOR] 4.82, 95% CI: 3.45–6.72)^31^. The authors concluded that the moments most at risk for HCPs may be outside the healthcare system (e.g., during home-to-work transport). A large case-control study in France identified that infected persons outside work are much more likely to play a role in SARS-CoV-2 transmission (aOR 19.9, 95% CI: 12.4–31.9), compared to infected colleagues (aOR 2.26, 95% CI: 1.53–3.33) or COVID-19 patients (aOR 2.37, 95% CI: 1.66–3.40)^32^. HCPs alternate between not wearing a mask (e.g., at home or during meals) and wearing a mask (e.g., during commute or at work), with varying degrees of compliance and exposure toward community transmission. During phases of high community transmission, the risk of infectious staff coming to work is high. Consequently, the benefit of the universal masking strategy in healthcare centers depends on the number of infected staff coming to work and also on the degree of compliance with mask-wearing by HCPs during their daily activities.

#### Protection of vulnerable and non-vulnerable patients

Patients in healthcare facilities are often vulnerable due to their age, comorbidities, and immunosuppression. Transmission from individuals with COVID-19 (patients, staff members, or visitors) is a relevant risk, especially during high prevalence of COVID-19 in the community. Before August 1, 2020, 11.3% (95% CI: 11.1–11.6) of hospitalized patients with COVID-19 in 314 UK hospitals got infected during hospital stay^33^. The risk of mortality was estimated to be 1.3 times higher in patients with hospital- compared to community-acquired COVID-19 (95% CI: 1.005–1.683)^34^. Among patients, vaccination largely reduces the risk of serious COVID-19. However, the risk associated with HA-RVI persists for unvaccinated individuals, with vaccine escape variants, or with specific underlying medical conditions^35^. Presenteeism, defined as working while being ill, is common among HCPs, even among those who work in high-risk settings^36^. HCPs coming to work with mild symptoms potentially expose patients and staff members, sometimes leading to clusters of infections among HCPs and patients. This risk increases with the virus activity in the community. Universal masking by all staff in the hospital might have a relevant benefit during high prevalence periods.

#### Protection of HCPs, prevention of absenteeism, and preservation of the hospital activity

RVIs are a major driver of absenteeism in the healthcare workforce during the winter season, when demand for healthcare services also peaks^37^. Absenteeism decreases the quality by limiting the capacity of care and by exposing the remaining professionals to extra workload and stress^38^. In a period of major staff shortages, absenteeism is a critical point to consider when reflecting on the use of PPE and recommending vaccination. Universal masking may represent a cost-effective strategy to address absenteeism, presenteeism, medical care, and mortality. However, maintaining HCPs with RVI at work with appropriate PPE in acute phases of staff shortage remains a concern.

The level of immune protection among HCPs is a critical factor to include in the evaluation of universal masking strategies. Although vaccination had an effect on transmission initially, now this effect has become limited. Still, vaccination prevents severe disease, and thus, has a benefit to the functioning of the healthcare system by shortening staff absence, allowing HCPs with mild symptoms coming to work, and keeping COVID-19 patients out of the hospital^39^. In a study performed in Greece, vaccination of HCPs reduced absenteeism from 11.8 to 4.7 episodes per 100 HCPs, and the duration of absence from 11.9 to 6.9 days during a period of high SARS-CoV-2 activity in the community^40^. Vaccination of HCPs against SARS-CoV-2 may protect patients from acquiring COVID-19^41^. However, the effectiveness of current COVID-19 vaccines at preventing healthcare-associated transmission remains uncertain, with some inconsistency across studies^42^. The viral load (which is linked to risk of transmission) of fully vaccinated and unvaccinated individuals was similar, suggesting comparable efficiencies of SARS-CoV-2 transmission^43^. In other reports, fully vaccinated individuals had a shorter duration of shedding of viable virus and a lower rate of secondary transmission than partially vaccinated or unvaccinated individuals^44^.

Despite long-term recommendations for influenza vaccination of HCPs, uptake remains low in most countries^45^. Regarding COVID-19 vaccination, on September 15, 2021, among 3 millions of HCPs in 2086 facilities in the US, 70.0% were fully vaccinated^46^. However, vaccine hesitancy in HCPs also occurred for COVID-19 vaccination before the winter season 2022/2023, and for the bivalent COVID-19 booster vaccine^47^. This may compromise the protection conferred at both individual and organizational levels. Offering influenza vaccination to staff translated into a 4.4% reduction in all-cause mortality in hospitals^48^. A modeling estimated that 100% vaccination coverage of HCPs could result in 43% reduction in the risk of infection in hospital patients and 60% reduction in nursing home patients^49^. According to these criteria, requiring unvaccinated HCPs to wear a mask when working in patient areas, and vaccine mandates for HCPs against COVID-19 and influenza must be discussed.

#### Availability, logistics, costs, and waste associated with face mask usage

The availability of good quality material, procurement, cost, and waste associated with the use of face masks are important factors to consider when deciding on universal masking as a prevention strategy. At the early stage of the pandemic, serious shortages of face masks were due to a combined increase in demand, shortage of production, and supply-chain ruptures. Production and supply-chain issues are now solved and most healthcare centers in high-income countries do not suffer from shortages anymore. However, the vulnerability of the system demands for stocking face masks in adequate storage conditions.

During the first year of the pandemic, the cost to procure PPE in hospitals in the US was estimated at more than $3 billion^50^. In 2019, hospitals spent around $7 per patient per day on PPE, $20.40 during spring 2020, and $12.45 during the height of the pandemic. Additionally, approximately 87,000 tons of PPE procured between March 2020 and November 2021 ended up as waste^51^. The COVID-19 waste story and the urgency to address sustainability also in healthcare ask for systems to reduce and manage healthcare waste including novel strategies of recycling and repurposing material.

### Alternative strategies for permanent universal masking in healthcare centers

Table 2 lists advantages and disadvantages of alternative strategies to permanent universal masking. The first level is symptom-based and includes transmission-based precautions as defined by standard precaution measures and respiratory etiquette. Face masks are used by HCPs during droplet exposure and by patients with respiratory symptoms. Symptomatic HCPs should be discouraged or not allowed coming to work. This strategy is promoted by groups of IPC specialists in US^52^. Asymptomatic and presymptomatic transmission is not addressed. The second level aims to protect patients and includes face masks in direct patient contact, either for all patients or for vulnerable patients only. HCPs wear masks during any activity with the patient. Patient-to-staff and staff-to-staff transmission is not addressed. The third level is epidemiology-driven and includes universal masking when disease activity in the community is high. The main gap in this strategy is the need for epidemiological data, either in form of a sentinel network or wastewater surveillance. The strategy is also challenged by the back-and-forth institution of universal masking depending on the epidemiology, which requires significant operational flexibility. The fourth level is a seasonal strategy, defining a time period for universal masking. Adherence with this policy by HCPs may be affected in case disease activity is still low in the community. The fifth level targets patient care and includes universal masking for all HCPs during their entire shift in all areas with patient care^53^. Adherence could be a real challenge outside periods of disease activity in the community. It also would generate a significant overuse of face masks raising cost-efficiency and ecological concerns. The last level aims at maximising risk reduction and includes permanent mask use by HCP, patients and visitors.

**Table 2.** Example of Advantages and Disadvantages of Alternative Strategies to Permanent Universal Masking in Healthcare Centers

Strategies	Description	Advantages of the strategy	Disadvantages of the strategy
Symptom-based precautions	Wearing a surgical mask in addition to standard precautions by patients with respiratory symptoms	- Better compliance with policy - Lower utilization of supplies - Better HCP-patient relationship	- Does not prevent asymptomatic and presymptomatic transmission - Requires high levels of vaccine and infection-induced immunity
Targeted masking	Wearing of a face mask in direct patient contact (either all patients or immunocompromised patients only)	- Better compliance with policy - Protection of (vulnerable) patients	- Does not prevent staff-to-staff transmission - Interferes with HCP-patient relationship
Epidemiology-based universal masking	Wearing surgical masks by all staff (clinical and nonclinical), patients, and visitors during high level of community transmission	- Adjustment to the risk of transmission, more acceptable by HCPs - Increased adherence and compliance with policy - Responsible utilization of supplies	- Difficult to implement in regions without sentinel data or wastewater surveillance - Challenge of back-and-force institution of a radical intervention in a complex environment
Season-based universal masking	Wearing a surgical mask by all staff (clinical and nonclinical), patients, and visitors during seasonal respiratory viral periods	- Adjustment to the theoretical risk of transmission of all respiratory viruses with a seasonal pattern - Takes into account the risk of asymptomatic and presymptomatic respiratory infections - Prevents hospital functioning	- Decreased adherence from HCPs during low level of community transmission - Not covering non-seasonal respiratory infections - Utilization of supplies
Targeted continuous masking	Wearing of a face mask by all HCPs during their entire shifts in areas with patient care	- Prevents HCP-patient and patient-patient asymptomatic and presymptomatic transmission - Increased adherence due to consistency of the strategy - Prevents presenteeism or absenteeism in clinical areas - Mitigates presenteeism in clinical areas - Preserves patient safety - Maintains clinical activity	- Utilization of supplies - Not preventing staff-to-staff transmission in nonclinical areas - Interferes with HCP-patient relationship
Permanent universal masking	Wearing a surgical mask by all staff (clinical and nonclinical), patients, and visitors at any time	- Prevents asymptomatic and presymptomatic transmission in the hospital - Prevents absenteeism - Mitigates presenteeism - Preserves patient safety - Maintains hospital activity	- Lack of adherence and compliance related to fatigue, discomfort and tolerability - Large utilization of supplies

Note. HCPs: healthcare professionals.

### Current masking strategies adopted in national and international guidelines

The US CDC adopted an epidemiology-based, targeted continuous masking strategy on September 23, 2022 (Table 3). Healthcare facilities in areas with low levels of community spread can “choose not to mandate” all clinicians, patients, and visitors to wear a face mask. Even if masking is not universally required, if a provider works in an area experiencing a COVID-19 outbreak, or if HCPs care for immunocompromised patients, they should wear a mask. When transmission levels are high, masking is recommended for everyone in a health care setting in areas with patients, but HCPs can choose not to wear masks when they are in “well-defined areas” that are restricted from patient access, like staff meeting rooms. The US CDC guidance shift created a controversy in the IPC world with professional societies urging to maintain mandatory mask requirement policies for healthcare employees in all patient care areas^54^. Among 44 healthcare epidemiologists in the US participating in a survey, 33 (97%) reported that their facility was maintaining universal masking despite CDC guidance, mostly (90%) for preventing non-SARS-CoV-2 seasonal viruses and to maintain staffing capacities (73%)^55^.

**Table 3.** Description of Masking Strategies for Source Control Adopted in National and International Guidelines

Country	Organization	Publication date	Guidelines content
International	WHO	Jan. 13, 2023	In areas of known or suspected community or cluster SARS-CoV-2 transmission: Universal masking: All health workers, including community health workers and caregivers, other staff, visitors, outpatients, and service providers, should wear a well-fitting medical mask at all times within the health facility and in any common area (eg, cafeteria, staff rooms). Inpatients not required to wear a mask unless physical distancing of at least 1 meter cannot be maintained or when outside of their care area In areas of known or suspected sporadic SARS-CoV-2 transmission: Targeted continuous medical mask use: health workers who work in clinical areas, should continuously wear a well-fitting medical mask during routine activities throughout the entire shift, apart from when eating and drinking. In non-patient areas, staff are not required to wear a medical mask during routine activities if they have no patient contact. No documented SARS-CoV-2 transmission: Medical mask use according to standard and transmission-based precautions
International	ECDC	Feb. 6, 2023	During periods of high community transmission of respiratory viruses such as SARS-CoV-2, influenza and RSV, Staff, visitors, and patients should be advised to wear medical face masks (universal masking) in common areas of the hospital, patient rooms, and other areas where patient care is provided. Alternatively, healthcare workers in contact with patients should wear a medical face mask during all routine patient care (targeted clinical masking). Universal and targeted clinical masking can be discontinued when the period of high community transmission is over. Decisions on the implementation of universal or targeted clinical masking should take into account the expected benefit, as well as the burden on resources, staff, patients, and visitors.
US	Center for Diseases Prevention and Control	Sept. 27, 2022	In all circumstances, source control recommended for individuals who: Have a suspected or confirmed SARS-CoV-2 infection or other respiratory infection (e.g., those with runny nose, cough, sneeze); Had close contact (patients and visitors) or a higher-risk exposure (HCP) with someone with SARS-CoV-2 infection, for 10 days after their exposure; Reside or work on a unit or area of the facility experiencing a SARS-CoV-2 outbreak; universal masking could be discontinued once no new cases have been identified for 14 days; High level of SARS-CoV-2 Community Transmission (≥ 100 New cases per 100,000 persons in the past 7 days at county level): Source control recommended for everyone in a healthcare setting in areas where they could encounter patients. HCP could choose not to wear source control when they are in well-defined areas that are restricted from patient access (eg, staff meeting rooms) Not high level of SARS-CoV-2 Community Transmission: Healthcare facilities could choose not to require universal source control. Individuals might also choose to continue using source control based on personal preference, informed by their perceived level of risk for infection based on their recent activities (eg, attending crowded indoor gatherings with poor ventilation) and their potential for developing severe disease.
England	UK Health Security Agency	Apr. 14, 2022	Standard precautions: worn (with eye protection) if a full-face visor is not available and spraying or splashing of blood, body fluids, secretions, or excretions onto the respiratory mucosa (nose and mouth) is anticipated or likely (Type IIR) Transmission-based precautions: Droplet precautions: Fluid resistant face mask should be put before entering the patient room/care area Airborne precautions: FFP3 infectious pathogen spread by the airborne route, and/or undertake aerosol generating procedures
France	French Society of IPC (SF2H)	Feb. 7, 2023	High level of SARS-CoV-2 Community Transmission (> 200 New cases per 100,000 persons in the past 7 days in the administrative department level) Universal masking for everyone entering the facility (indoor and outdoor) Moderate level of SARS-CoV-2 Community Transmission (11 to 200 New cases per 100,000 persons) HCP: Masking in every care situation and face to face situations with patients inside the facility (indoor). In non-patient areas, staff are not required to wear a medical mask during routine activities if they have no patient contact. Continuous masking when signs of respiratory infection Patients: masking when entering in the facility (indoor), when someone entering in the room, or when getting out of the room Low level of SARS-CoV-2 Community Transmission (≤ 10 New cases per 100,000 persons) HCP: during care at risk of body fluid exposure, and when signs of respiratory infection Patients: only when signs of respiratory infection
Switzerland	Swissoso	Jan. 31, 2023	Patients: mandatory for all patients with respiratory symptoms HCPs: mandatory for all HCPs with respiratory symptoms; mandatory in direct patient contact depending on local epidemiology Visitors: recommended when entering patient rooms or otherwise direct patient contact depending on local epidemiology Universal masking can be considered depending on local epidemiology and in case of staff shortage

Note. RSV, respiratory syncytial virus; HCP, healthcare professionals.

On January 13, 2023, WHO released an update of the living IPC guideline taking into account the evolving epidemiological trends for COVID-19. The masking strategy was adapted to the SARS-CoV-2 transmission with three levels^56^. When any SARS-CoV-2 transmission is documented in the area, medical masks can be used according to standard and transmission-based precautions. In areas of known or suspected sporadic SARS-CoV-2 transmission, the strategy is based on a targeted continuous medical mask use. In areas of known or suspected community or cluster SARS-CoV-2 transmission, universal masking applies for HCPs but not for patients unless physical distancing of at least one meter cannot be maintained.

The ECDC guidelines, published on February 6, 2023, recommend universal masking for staff, visitors, and patients in common areas of the hospital, patient rooms, and other areas where patient care is provided, during periods of high community transmission of respiratory viruses such as SARS-CoV-2, influenza, and RSV^57^. Alternatively, targeted clinical masking is recommended for HCP in contact with patients during all routine patient care.

In Switzerland, the strategy is epidemiologically-driven with mandatory masking for all HCPs in direct patient contact or universal masking depending on local epidemiology and in case of staff shortage^58^.

Since February 7, 2023, SARS-CoV-2 community transmission is guiding the masking policy in France^59^. Universal masking is mandatory for every individual entering the facility (indoor and outdoor) when >200 new cases per 100,000 population have been reported during the past seven days in the administrative department. During phases of moderate transmission (11 to 200 new cases per 100,000 population), a targeted continuous masking is adopted in patients-areas, with a flexibility to not wear the mask in non-patient areas. Standard and transmission-based precautions are applied ≤ 10 new cases per 100,000 population. In England, since May 27, 2022, the national IPC manual reverts back from universal masking to standard and transmissions-based precautions^60^.

## Conclusion

In conclusion, three years after the emergence of the COVID-19 pandemic and the improved understanding of the transmission of RVI, the IPC community should consider revisiting the current masking policies. Specifically, de-escalation of the universal masking strategy implemented in healthcare centers since the first wave of COVID-19 must be revisited. The current strategies by most national and international organizations are based on the level of community transmission of SARS-CoV-2. Actions are now required to establish a list of the most burdensome RVI to consider, implement reliable local surveillance of RVI, and define thresholds for activating masking strategies. Considering the epidemiological context (measured via existing sentinel networks or innovative ways such as wastewater analysis), and if not available considering a time period (winter season) may guide to three gradual levels of masking: (i) standard and transmission-based precautions and respiratory etiquette, (ii) consistent face mask wearing when in direct contact with patients, and (iii) universal masking. Cost-effectiveness analyses are warranted in the coming years to refine recommendations. The face mask is only one element among other preventive measures in healthcare settings such as staff and patient immunization, and efficient ventilation.
