# Considerations for de-escalating universal masking in healthcare centers – CORRIGENDUM

**DOI:** 10.1017/ash.2023.438

**Published:** 2023-09-15

**Authors:** Gabriel Birgand, Caroline Landelle, James R. Price, Nico T. Mutters, Daniel J. Morgan, Jean-Christophe Lucet, Solen Kerneis, Walter Zingg

In the above published article^1^, the authorship should appear as follows:

Gabriel Birgand, Caroline Landelle, James R. Price, Nico T. Mutters, Daniel J. Morgan, Jean-Christophe Lucet, Solen Kerneis and Walter Zingg
